# High Burden of Non-Clonal Chromosome Aberrations Before Onset of Detectable Neoplasia in Fanconi Anemia Bone Marrow

**DOI:** 10.3390/cancers17111805

**Published:** 2025-05-28

**Authors:** Silvia Sánchez, Benilde García-de-Teresa, Marco A. Mejía-Barrera, Pedro V. Reyes-Jiménez, Antonio Paz-Martínez, Miguel A. Martínez, Moisés Ó. Fiesco-Roa, Angélica Monsiváis-Orozco, Bertha Molina, Leda Torres, Alfredo Rodríguez, Sara Frias

**Affiliations:** 1 Laboratorio de Citogenética, Instituto Nacional de Pediatría, Insurgentes Sur 3700-C, Ciudad de México CP 04530, Mexico; ssanchezs@pediatria.gob.mx (S.S.); b.garciadeteresa@gmail.com (B.G.-d.-T.); marcoamb1989@gmail.com (M.A.M.-B.); reyesj87@gmail.com (P.V.R.-J.); antonio_paz@ciencias.unam.mx (A.P.-M.); fiescoroa@facmed.unam.mx (M.Ó.F.-R.); bertha_molina@yahoo.com.mx (B.M.); ledactorres@gmail.com (L.T.); 2 Posgrado en Ciencias Biológicas, Universidad Nacional Autónoma de México, Ciudad Universitaria, Ciudad de México CP 04510, Mexico; 3 Servicio de Hematología, Instituto Nacional de Pediatría, Insurgentes Sur 3700-C, Ciudad de México CP 04530, Mexico; angelicamonsivais@hotmail.com; 4 Laboratorio de Falla Medular y Carcinogénesis, Instituto Nacional de Pediatría, Insurgentes Sur 3700-C, Ciudad de México CP 04530, Mexico; alfredo.rodriguez@iibiomedicas.unam.mx; 5 Departamento de Medicina Genómica y Toxicología Ambiental, Instituto de Investigaciones Biomédicas, Universidad Nacional Autónoma de México, Ciudad Universitaria, Ciudad de México CP 04510, Mexico

**Keywords:** Fanconi anemia, non-clonal chromosome aberrations, clonal chromosome aberrations, complex karyotypes, cancer evolution, bone marrow failure, AML, MDS

## Abstract

**Background/objectives:** Fanconi anemia (FA) is an inherited bone marrow failure syndrome characterized by chromosome instability and predisposition to develop myelodysplastic neoplasm (MDS) and acute myeloid leukemia (AML). Clonal chromosome aberrations (CCAs) in chromosomes 1, 3, and 7 frequently appear in the bone marrow (BM) of patients with FA and are associated with MDS/AML progression. Given the underlying DNA repair defect that characterizes FA, non-clonal chromosomal abnormalities (NCCAs) are expected to be common events in the FA BM; in this study, we investigated the presence and significance of NCCA and CCA in the bone marrow (BM) of patients with FA. **Methods:** Here, we transversally examined the BM karyotypes of 43 non-transplanted patients with FA, 41 with non-clinically detectable hematologic neoplasia and two with diagnosed MDS. We searched for the presence of NCCAs, complex karyotypes (CKs), and CCAs as well as their association with the natural history of the disease, including age, degree of BM failure, and neoplastic transformation. **Results:** NCCAs were observed in the metaphase spreads of 41/43 FA patients; CKs were observed in 25/43 patients; CCAs were found in 15/43 patients; CCAs involving chromosomes 1, 3 and/or 7 were found in four patients; and other autosomes were found in the remaining 11 patients. Overall, we observed a baseline large karyotypic heterogeneity in the BM of FA patients, demonstrated by the ubiquitous presence of NCCA; such karyotypic heterogeneity precedes the eventual emergence of CKs and selection of cells carrying fitness-improving CCAs. Finally, CCAs involving chromosomes 1, 3 and 7, well-known drivers of hematological malignancy in FA, become established. Overall, we observed that the frequency of NCCAs and CCAs increased with age, even though a significant correlation was not found. **Conclusions:** These observations fit the model of evolution towards cancer that comprises a first phase of macroevolution represented by NCCAs and karyotypic heterogeneity, followed by the establishment of clones with CCAs, leading to microevolution and cancer. NCCAs are the most frequent chromosomal alterations in the bone marrow of patients with AF and constitute a genome with extensive karyotypic heterogeneity that evolves into clones with more complex genomes and can eventually progress to cancer.

## Simple Summary

Fanconi anemia (FA) is the most common inherited bone marrow failure syndrome, characterized by chromosomal instability and a high risk of cancer. Hematologic malignancy in FA, mainly myelodysplastic neoplasm (MDS) and acute myeloid leukemia (AML), associates with characteristic clonal chromosomal abnormalities in the bone marrow. Although clonal chromosome abnormalities (CCAs) associated with malignant progression in FA involve chromosomes 1, 3, and 7, information on the broader preleukemic bone marrow cytogenetic landscape is scarce. In this study, we report the type and frequency of every kind of non-clonal chromosomal abnormality (NCCA) appearing in the bone marrow of a group of patients with FA, spanning from cancer-free patients to hematologic malignancy, where CCA appears. This study unveils the dynamism of emerging karyotypes in the FA bone marrow and its potential association with the patient’s hematological state.

## 1. Introduction

Fanconi anemia (FA) is a chromosome instability syndrome affecting 1 to 5 individuals per 1,000,000 individuals. FA is also the most frequently inherited bone marrow failure syndrome and originates from the inheritance of pathogenic variants (PVs) in one of any 23 genes (FANCA-FANCX) that operate in the FA/BRCA pathway, responsible for recognizing and repairing DNA interstrand cross-links (ICL), through homologous recombination, an error-free pathway of DNA repair [1,2,3]. In the absence of a functional FA/BRCA pathway, the ICLs can be repaired by low-fidelity DNA repair pathways, such as the non-homologous end joining (NHEJ) or the microhomology-mediated end joining (MMEJ) pathways [4,5], which promote cell viability at the expense of increasing chromosome instability (CIN). The latter is a pathognomonic feature of FA, which allows its diagnosis due to the sensitivity of FA cells to ICL-inducing agents, like mitomycin C (MMC) and Diepoxybutane (DEB) [2,6,7,8,9].

The FA clinical phenotype is highly heterogeneous, affecting multiple organs and systems. The phenotype includes developmental abnormalities that can be detected from birth and that are present in 80–96% of patients [10,11]. Bone marrow (BM) failure, which appears in over 86% of patients with FA, consists of aplastic BM accompanied by peripheral blood cytopenia of different degrees: mild, moderate, or severe; patients with FA will develop BM failure around 7 years of age [11,12]. Also, FA patients are prone to develop cancer, both hematologic and solid tumors, with a cumulative incidence by 40 years of age of 30–33% and 20–28%, respectively [13,14]. The relative risk for a patient with FA to develop cancer with respect to the general population is 19X and rises post-HSC transplant to 55X; specifically, 527X for head and neck squamous cell carcinomas (HNSCC), and after transplant, 933X; 582X for vulvar squamous cell carcinomas, and after transplant, 6298X; 213X for acute myeloid leukemia (AML); and 5669X for myelodysplastic neoplasia (MDS). The risk for hematological cancer, MDS and AML, increases by the age of 10 years old, plateauing at 20–30 years old [14,15].

BM failure in FA has been proposed to be the consequence of damage accumulation in the DNA of hematopoietic stem and progenitor cells (HSPCs) and hyperactivation of growth suppressing pathways, such as the TGFβ (Transforming growth factor β) and the p53/p21 pathways, thus resulting in critical reductions in the number of HSPCs [16,17]. Despite this harsh BM microenvironment, certain HSPCs thrive, probably due to the inherent CIN in FA and the acquisition of chromosome abnormalities that allow temporary survival, although not in the long term.

CIN, one of the hallmarks of cancer, is characterized by aneuploidy and structural chromosomal abnormalities (CAs) and can be observed in the early stages of cancer in the general population [18,19,20]. Importantly, CIN is a constant in FA patients; from zygote formation and embryonic development, it produces single cell genomic variation in the form of heterogeneous karyotypes and has the potential to produce, across the body of patients with FA, diverse altered genomes with short-term survival and elevated level of cell death, leading to the loss of somatic and HSPS cells [21,22]. This karyotypic heterogeneity and its inherent proapoptotic tendency might also be the foundation for the well-known appearance of clonal hematopoiesis and eventual progression to cancer in FA [22,23]. The latter might be the reason why up to 40% of children and young patients with FA show signs of clonal evolution in their BM, and 15 to 60% will develop MDS or leukemia, mainly AML, at an early age [2,24].

Multiple studies have described, and confirmed, the involvement of clonal chromosome abnormalities (CCAs) in the emergence of MDS or AML in patients with FA [23,24,25,26,27,28]. Most of these studies screened for common CCAs using microarrays or fluorescent in situ hybridization (FISH) directed to regions of interest in chromosomes 1q, 3q, and 7q, since these are the most informative cytogenetic markers of clonal evolution towards MDS/AML [24,29,30]. On the other hand, non-Clonal Chromosome Aberrations (NCCAs) are often overlooked in cytogenetic analysis because they appear in only a single cell and are dismissed as “background noise” [31], especially in patients with CIN, as is the case for FA [24].

NCCAs involve large-scale genomic changes, including numerical and structural chromosomal abnormalities resulting in the gain and loss of genomic material; they also include balanced structural alterations such as translocations or inversions, which modify the topology of chromosome segments and change the gene interaction at the scale of an entire genome [32], leading to a karyotypically diverse cell population, with each cell potentially having unique evolutionary properties. NCCAs have gained importance because they are known to alter the genomic information system by changing the karyotype coding, either through a loss or gain of genetic material or by changes in the topology of genes and their regulatory elements, or both. Thus, each cell with a specific NCCA has unique evolutionary potential, and this karyotypic heterogeneity of a cell population is essential for macroevolution toward disease, and specifically, toward cancer [33].

Cancer evolution is known to be a dynamic process, directed by large-scale chromosomal copy number variations that create karyotypic heterogeneity between the cells that compose a tissue, privileging the selection of the genomes with the greatest fitness [18] and eventually leading to the selection of clones with CCAs that will orchestrate cancer development and evolution [18,34]. FA-inherent CIN is expected to create large levels of NCCA, generating a BM with high karyotypic heterogeneity, years before full-blown MDS or AML. In this sense, NCCA could precede the formation of complex genomes and stable CCAs, eventually transitioning to cancer.

The aim of this study was to cytogenetically analyze the BM of a group of non-transplanted patients with FA, to investigate whether the multiple steps of karyotypic evolution towards cancer can be considered in relation to the age of the patients and their hematological condition. We did not find an association between the complexity of the BM karyotype and the age of the patients or their hematological condition, suggesting that the age and stage of bone marrow failure are not the only drivers of leukemogenesis in FA. However, inspecting the group of FA patients as a whole revealed transitions from a karyotypically diverse population of BM cells with NCCA to the presence of complex karyotypes and the emergence of CCAs involving CA of indeterminate potential in patients with FA [30] and CCAs with the classic high-risk chromosome alterations found in chromosomes 1, 3, and 7; the only two patients in the group who were identified with MDS presented with the classic alterations in chromosomes 3 and 7.

## 2. Materials and Methods

### 2.1. Patients

We performed a transversal study including non-transplanted patients with a confirmed diagnosis of FA with the DEB chromosome breakage test. The samples analyzed corresponded to the first cytogenetic BM evaluation of each patient within the framework of the recommended annual follow-up in the search for MDS/AML progression markers. All samples were processed at the Cytogenetics Laboratory of the National Institute of Pediatrics (Mexico) between 2015 and 2024. In accordance with the Declaration of Helsinki, all patients or their relatives gave written informed consent for genetic analyses under project numbers INP 2014/041 and 2020/012; of the 43 patients studied, 33 had available genotyping data. The distribution of PVs among *FANC* genes in this cohort is similar to that reported in other studied populations. As expected, the most frequently mutated gene was *FANCA*. Variants in genes other than *FANCA* showed slight differences in frequency compared to those reported in previous studies [11,35]. Among the 33 genotyped patients, 22 (67%) had a PV in *FANCA*. The remaining 33% were distributed as follows: 3/33 in *FANCE*, 2/33 in *FANCG*, 2/33 in *FANCL*, and 1/33 each in *FANCF*, *FANCD2*, *FANCJ*, and *FANCN*. The available genetic information for FA is presented in Supplementary Table S2. Clinical and demographic data were obtained from the patient’s medical records and interviews conducted under the framework of the Fanconi Anemia Registry from Mexico (RAFMex), project INP 2020/053. These projects were approved by the institutional research and ethics committees at Instituto Nacional de Pediatría (México).

Age at diagnosis was calculated considering the date of a positive DEB test. BMF severity at the time of cytogenetic study was classified according to Fanconi anemia guidelines for diagnosis and management [35], using the hemogram data (free of transfusion) nearest to the cytogenetic study. The use of androgens was recorded irrespective of the length of treatment or if being taken at the time of sample collection. Transfusion dependency was considered as the time when the patient could not maintain adequate blood counts without sequential transfusions at any timepoint.

### 2.2. Cytogenetics

Conventional karyotypes were performed on BM cells using the standard direct method. Briefly, 1–2 mL of heparinized BM samples were incubated in a 5% CO_2_ incubator, cultured during 48 h in MarrowMax medium (Gibco, Life technologies, Grand Island, NY, USA) without cell division stimulant at 37 °C, after which colcemid [10 μg/mL] (Gibco, Life technologies, NY, USA) was added for the final 2 h of culture. Harvesting, slide preparation, and GTG banding were conducted according to the classic methodology [36]. On average, 20 metaphase spreads were analyzed per patient and reported according to the International System for Human Cytogenetic Nomenclature (ISCN) [37]; fewer than 18 metaphases were analyzed in four patients with severe aplastic anemia due to hypocellular BM.

Chromosome abnormalities were classified as (a) NCCA, non-clonal chromosomal abnormality, both numerical and structural; (b) CK, complex karyotype, defined in this study as a cell with ≥3 independent cytogenetic abnormalities [38]; (c) CCA, clonal chromosomal abnormalities, when at least two cells bear the same chromosome gains or structural rearrangements (including deletions and duplications) or when at least three cells present with the same whole chromosome loss [37]. CCA 1,3,7 refers to clonal abnormalities involving at least one of the known recurrent alterations, i.e., duplication of the long arm of chromosome 1 (commonly known as 1q+), duplication of the long arm of chromosome 3 (commonly known as 3q+), complete monosomy of chromosome 7 or deletion of the long arm of chromosome 7 (commonly known as –7/7q–), while CCA refers to clonal chromosome alterations in chromosomes other than 1q+,3q+ and –7/7q–. When aneuploidies of a whole chromosome were detected at a given metaphase, and to reduce potential technical errors, the periphery of such metaphases was analyzed with a 10Xobjective to increase the visibility margin by 10 fields and to detect whether the lost chromosome was located at the periphery of the metaphase or whether the gained chromosome belonged to a nearby metaphase. Chromosome breaks and radial exchange figures were recorded during the screening but are not part of the analysis in this report.

### 2.3. Statistics

All statistical analyses were performed in Prism (version 10.4.0). Normal distribution of the data was assessed with the Shapiro-Wilk test. Data with normal distribution were compared using one-way ANOVA with Tukey’s post-test for multiple comparisons. Comparisons between two groups were performed with the unpaired t test. The Fisher’s exact test was used to compare proportions between two groups. The Pearson correlation test was used to probe correlation between age and frequency of chromosome abnormalities. The log-rank (Mantel-Cox) test was used to compare the survival of the patients, according to the presence of different types of CA.

## 3. Results

### 3.1. Patients

Samples from 50 non-transplanted patients with a positive diagnosis of FA were sent to the cytogenetics laboratory at Instituto Nacional de Pediatría (Mexico) for routine annual follow-up between 2015 and 2024; six samples had insufficient material to perform cytogenetic analysis, and one patient was excluded since the provided sample was obtained once antineoplastic treatment for AML was started. Appropriate cells for cytogenetic analysis, in quality and number, were obtained from 43 patients (Table S1); in two of these (FANC031 and RAFMex015), the concurrence of cytopenia and a monosomy of chromosome 7 clone allowed the diagnosis of MDS in accordance with International Consensus Classification criteria [39]. Over the course of this study, some of the patients received more than one cytogenetic analysis on more than one sample; only the first cytogenetic study was considered for the purposes of this report.

Median age at diagnosis was 8.1 (1.4–15.6) years old, and median age at the time of the cytogenetic study was 9.14 (4.7–28.6) years old, with a female-male relationship of 1.4:1. A summary of the population demographics is shown in Table 1, and the main clinical data are shown in Table 2. Eleven patients died during the time that elapsed between the cytogenetic study and this report, due to BM failure or HSCT-related complications in eight patients, MDS in two patients, and acute lymphoblastic leukemia (ALL) in the remaining one.

### 3.2. BM Cytogenetics of Patients with FA

**Figure 1:**
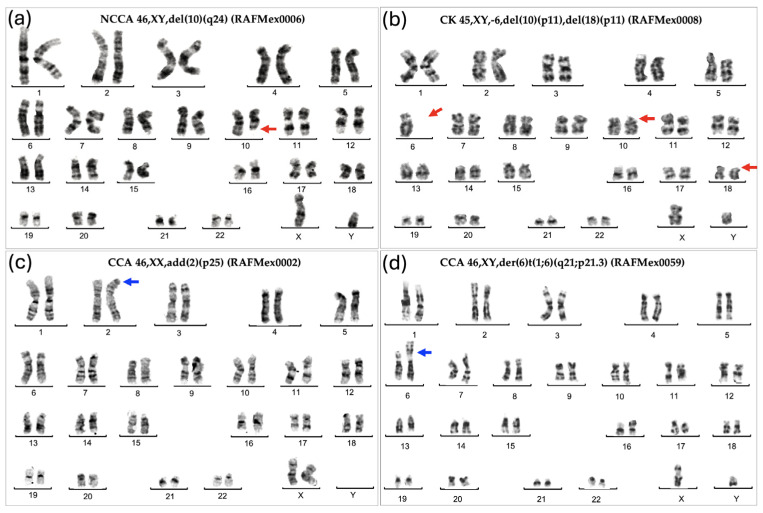
Representative karyotypes showing the various types of chromosomal abnormalities found in the BM from patients with FA. (**a**) NCCA: Non-Clonal Chromosome Aberration; (**b**) CK: Complex Karyotype showing monosomy of chromosome 6 and deletion of 10p and 18p; (**c**) CCA = Clonal Chromosome Aberration of indeterminate potential; (**d**) Clonal Chromosome Aberration involving the duplication of the region 1q, a high risk chromosomal abnormality. Red arrows indicate deletions; blue arrows indicate duplications.

As expected for patients with FA, their BM showed enormous karyotypic heterogeneity, evidencing the underlying CIN. The cytogenetic analysis in the 43 included patients showed that all but one patient displayed chromosomal abnormalities, which were classified in three types: NCCA, non-recurrent CK, and CCA. Representative karyotypes per CA type are shown in Figure 1.

**Figure 2:**
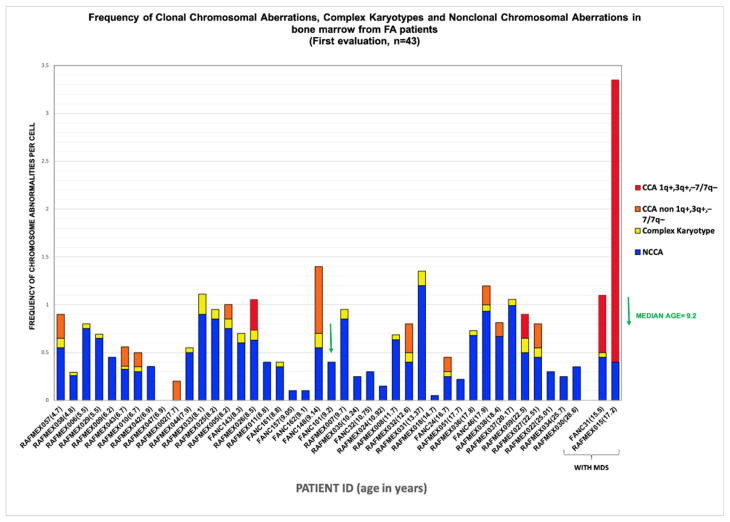
Frequency of chromosomal aberrations found in BM of FA patients ordered by age and MDS diagnosis at time of cytogenetic study. Distribution of the frequencies of the different type of chromosomal aberrations, per patient, in ascendent order of age. Age is indicated in parentheses.

**Figure 3:**
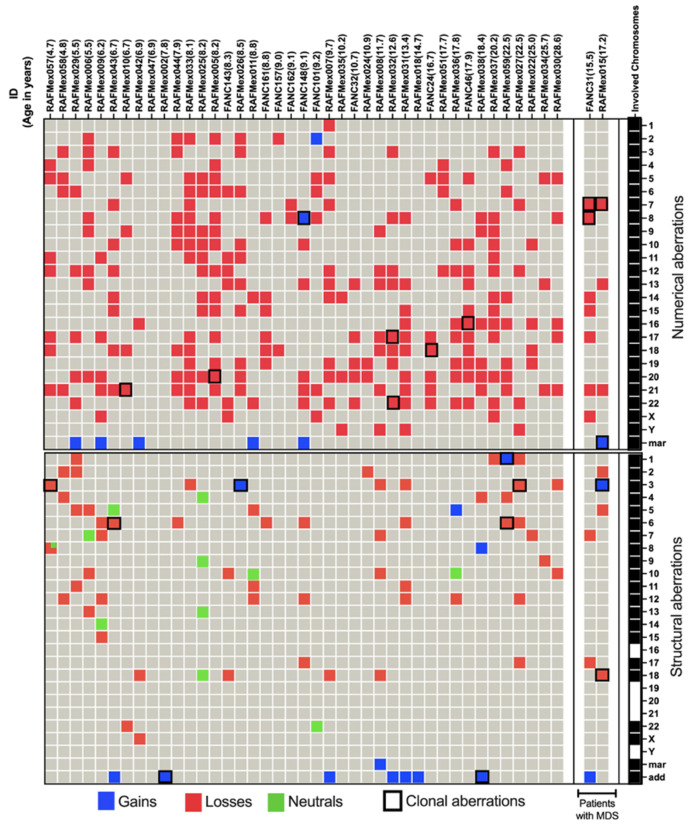
NCCA and CCA abnormalities in 43 FA patients. In the upper panel, patients are ordered by age and MDS diagnosis. The rightmost column signals which chromosomes are involved in CA. Complete aneuploidy, including loss and gain, was observed for all 24 chromosomes. Structural aberrations, depicted in the lower section, involved all chromosomes except 16, 19, 20, 21, and Y. Marker chromosomes refer to non-identified abnormal chromosomes and add refers to additional chromosomal material of unknown origin. Neutral aberrations signal inversions and balanced translocations. Patient RAFMex057 showed two structural alterations on chromosome 8, one deletion and one inversion, as indicated by 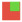.

NCCAs were observed in 41/43 patients; karyotypes where NCCAs were the only findings occurred in 26/43 patients; the presence of only CCA was observed in a single patient; and the combination of both NCCA and CCA was observed in 15/43 patients. In addition, 25 patients presented with non-recurrent CK. Among the 15 patients with CCA, eight had clones with aneuploidy for one complete chromosome, six had clones with structural chromosomal aberrations, and one patient had clones with both types of chromosomal aberration (Figure 2). Chromosome losses were more commonly observed in comparison to chromosome gains. The chromosomes more frequently involved in any kind of CA were chromosomes 3, 7, 8, and 18, followed by chromosomes 6, 16 12, 17, 20, and 22; also, we found additional material (add), marker chromosomes (mar), and neutral aberrations like inversions and balanced translocations (Figure 3 and Table 3).

The complexity and heterogeneity of the karyotypes present in the bone marrow (BM) of patients with Fanconi anemia (FA) are evident in Figure 2 and Figure 3 and Table 3. As shown, all but one patient (RAFMex047) presented with composite karyotypes. Unlike a classic karyotype, in which no CAs are found or in which the same alteration is found in all cells, we refer to a composite karyotype as a karyotype in which multiple chromosomal changes, both numerical or structural, are identified in different cells, meaning there is cell-to-cell variation in CA within the same cell population [40], forming a “composite” genetic profile. Specifically in neoplasias, the composite karyotype is useful to track how chromosomal alterations change in a cell population over time by evidencing the karyotypic heterogeneity of the clones, where the metaphases are different, although they share CCA due to the accompanying alterations, which can subsequently form subclones such that clonal evolution is evident [37]. We found composite karyotypes in patients with and without clones, reflecting a highly complex cell population harboring distinct chromosomal alterations, primarily copy-number variations, which underscore the karyotypic diversity resulting from chromosomal instability in patients with FA [23,37].

### 3.2.1. Non-Clonal Chromosomal Abnormalities

Patients with inherent CIN, such as in FA, are expected to have enormous karyotypic heterogeneity, as was evidenced in their BM, where stochastic chromosomal alterations were found (Table 3 and Figure 2). All 24 human chromosomes were involved in these CAs, and numerical aberrations were the most common (Figure 3).

NCCAs were observed in the vast majority of FA patients, 95% (41/43), including non-recurrent CK. NCCAs without evidence of any additional chromosomal clones were found in 26 patients. NCCAs were the most prevalent type of CA, accounting for 74.6% of the total abnormalities observed, and included CK in 25/43 patients, with most being non-recurrent. Patient RAFMex015 was exceptional, since clonal and complex karyotypes were found in 100% of the analyzed cells, concurrent with an MDS diagnosis (Table 3 and Figure 2).

In most patients, NCCAs were the most frequent CAs; these were highly diverse and included numerical and structural alterations. Among the structural CAs, 11/78 were neutral in that they did not appear to condition a loss or gain of chromosome information; most were inversions, but there was also one translocation (Figure 3, green cells).

### 3.2.2. Clonal Chromosomal Abnormalities (CCAs)

CCAs accounted for 25.4% of the total CAs found in the 43 patients with FA (Table 3). They were found in 35% (15/43); among these, the presence of CCAs involving monosomy of chromosome 7 in two of these patients led to a MDS diagnosis, and both presented with a composite karyotype, RAFMex015: 45,XY,–7,–18,+der?(18)t(3;18)(q13.3;q?12)[15]/45,idem,del(2)(p23)[1]/45,idem,del(5)(p14)[1]/45,idem,–13,+mar2[1]/45,idem,–21,+mar1[2][cp20]. In this karyotype, a main stem line with a clone with monosomy 7 and a subclone with monosomy 21 were found; both alterations were recognized as high risk in patients with FA, with an evident clonal evolution. The patient FANC31: 45,XX,–7[8]/44,sl1,add(1)(p36),–17[1]/45,XX,–8[2]/44,sl2,–15[1]/45,X,–X[1]/44,XX,del(7p),–17,–21[1]/45,XX,–14[1]/46,XX,del(17)(p11.2)[1][cp16]/46,XX[4], presented with two unrelated stem lines, one of them -sl1- with monosomy 7, also a high-risk CA. Within the 15 patients that presented with clonal chromosome abnormalities (WC), seven presented clones consisting of whole-chromosome aneuploidy, losses involving chromosomes 7, 8, 16, 17, 18, 20, 21, and 22, gains involving chromosome 8, and/or a marker chromosome. Seven patients had clones with structural CA in chromosomes 1, 3, 6, and 18, and one patient presented a clone with both numerical (chromosomes 7 and +mar) and structural (chromosomes 3 and 18) CAs. Among 15 patients WC, 11 presented CCAs involving autosomes other than 1, 3, and 7, and in 4/15 patients WC, the clone involved the regions on chromosomes 1, 3, and 7 associated with the evolution towards hematological malignancy; one of them had a duplication 1q21-qter, due to a translocation t(1;6)(q21;p21.3). Patients WC, in addition to having cytogenetic clones, revealed a variety of NCCAs: 3/15 had whole-chromosome aneuploidies, 11 had numerical and structural NCCAs, and only one patient did not present NCCAs (Figure 3 and Table 3). Within the chromosomes or chromosomal regions involved in CCAs, in addition to the genes commonly associated with MDS and AML in patients with FA, such as MDM4, EVI1, and RUNX1 [23,26], we identified other genes contained in CCAs that may also play a role in the progression to these malignancies, as detailed in Supplementary Table S3.

### 3.3. Clonicity and Chromosomal Damage

**Figure 4:**
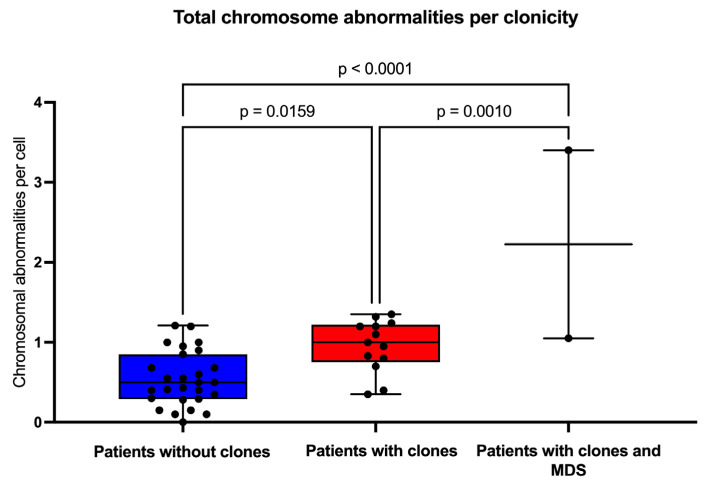
Frequency of chromosomal aberrations per cell. The population was divided into patients without clones (blue box), with clones (red box), and with –7/MDS (myelodysplastic neoplasm). Statistical analyses were performed using one-way ANOVA with Tukey’s post-test for multiple comparisons.

In this group of patients, we find that the average frequency of CCAs (0.16) is only ~20% of the observed average frequency of NCCAs (0.47) (Table 3). In addition, it is evident that when the total frequency of ab/cell is higher, CCA appears in a patient (Figure 4). In patients WC who have MDS, a large number of CAs are observed in their BM, because the fittest clone is present in a large proportion (Figure 1 and Figure 4). In patients WC, CCA alterations coexist with NCCAs, although the latter are generally the most common alteration. However, it should be noted that in the two patients who already presented MDS, CCAs prevailed over NCCAs (Figure 2).

### 3.4. Complex Karyotypes and Clonicity

**Figure 5:**
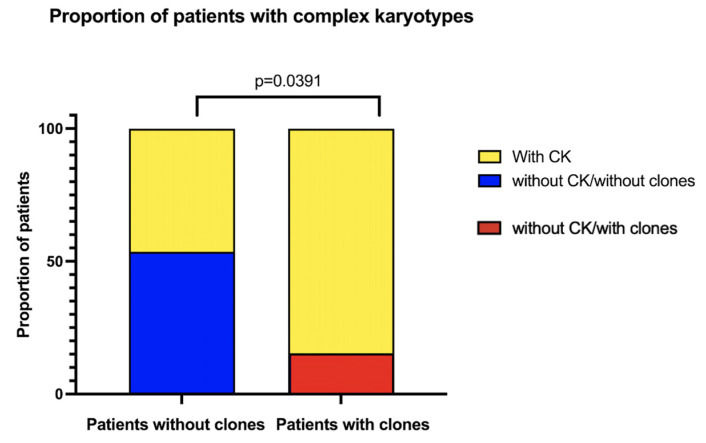
Patients with complex karyotypes (CKs), according to clonicity. In yellow, the proportion of patients who present at least one metaphase with CKs (≥3 CA). Patients with MDS were excluded.

Several analyzed metaphases had more than one CA; we classified the patients that had at least one metaphase that also had three or more CAs as having a CK. Twenty-five out of 43 patients had metaphases with these CKs, which represent a greater complexity within the population of cells with NCCAs. When comparing the frequency of CKs in patients with and without clones, there is a higher proportion of patients with CKs among those who have already developed clones, showcasing an increased complexity of the diverse karyotypes found in their BM (Figure 5).

### 3.5. Chromosomal Damage and Patient Demographic and Clinical Characteristics

#### Chromosomal Abnormalities and the Age and Gender of Patients

**Figure 6:**
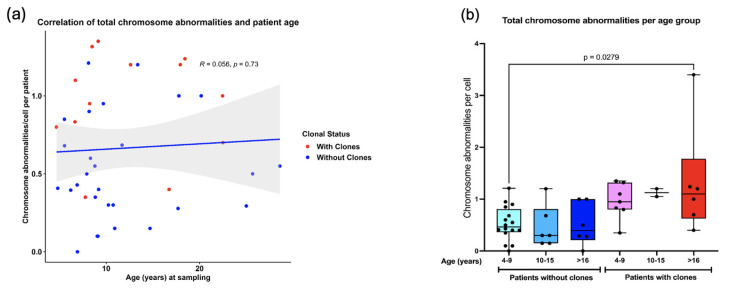
Correlation between the total frequency of CAs and patient age. (**a**) No correlation was found between the amount of CAs and increasing age of patients WC and without clones (Pearson R = 0.056 *p* = 0.73). (**b**) A significant increase in total CAs was found only in patients 4–9 years old without clones and patients > 16 years with clones.

**Figure 7:**
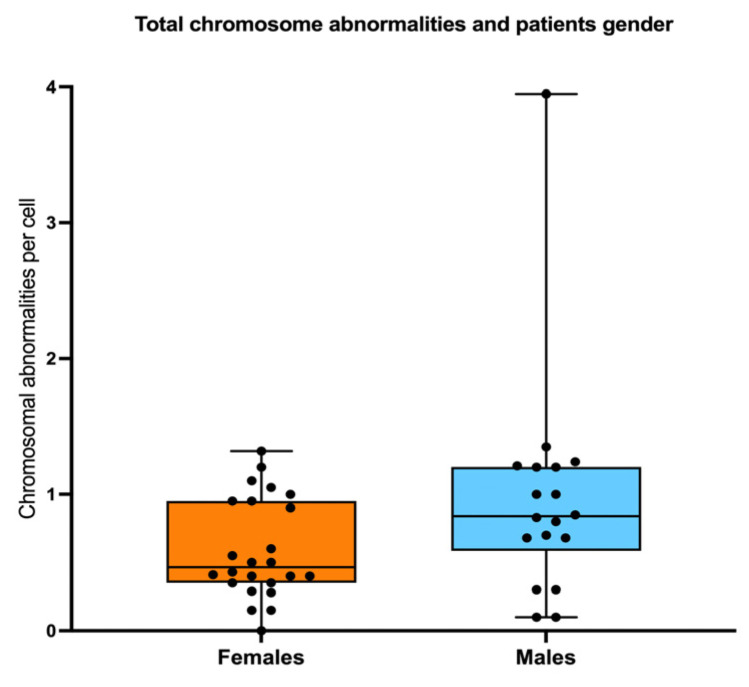
Sex does not influence the frequency of CAs. No differences in the total frequency of CAs were found when comparing females vs. males.

Pearson correlation analysis revealed that as the patients grow older, the total frequency of CAs does not increase (R = 0.056, *p* = 0.73) (Figure 6a). However, when patients were sub-divided into three groups of age, 4–9 years old, 10–15 years old, and >16 years old, a significantly higher frequency of CAs was observed between the older patients WC (excluding MDS patients) and the younger patients without clones (one-way ANOVA with Tukey’s post-test) (Figure 6b). Finally, there were no significant differences in AC frequency when comparing males and females (Figure 7).

**Figure 8:**
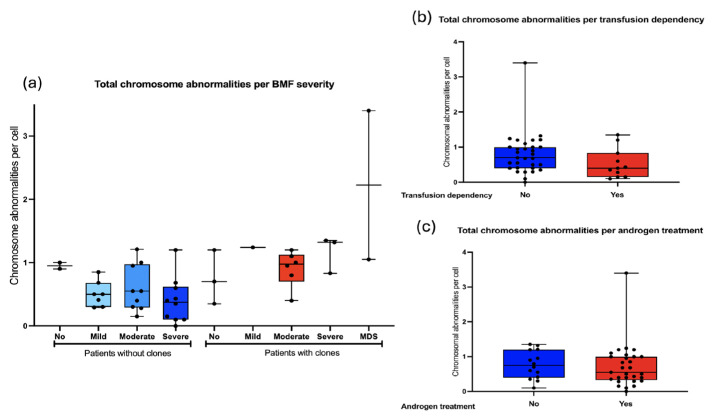
Frequency of CA with respect to hematologic status. (**a**) The frequency of CA in FA does not correlate with the severity of bone marrow failure, determined in accordance with the FA clinical care guidelines [35]. (**b**) CA per cell with respect to transfusion dependency. (**c**) CA per cell in reference to androgen therapy. No differences in the frequency of CA were observed with respect to hematological conditions or patients’ treatment.

We searched for an association between the frequency of chromosomal aberrations and bone marrow failure severity: 5/43 did not have BMF, 28/43 had moderate or severe BMF. As a group, the patients WC show slightly more CA than WOC, and although no statistical differences were found, it would appear that in individuals WC, CAs would increase as the BMF becomes more severe (Figure 8a). Other hematological status parameters, including transfusion dependency and androgen treatment, did not influence the total frequency of CAs observed in patients with FA (Figure 8b,c).

**Figure 9:**
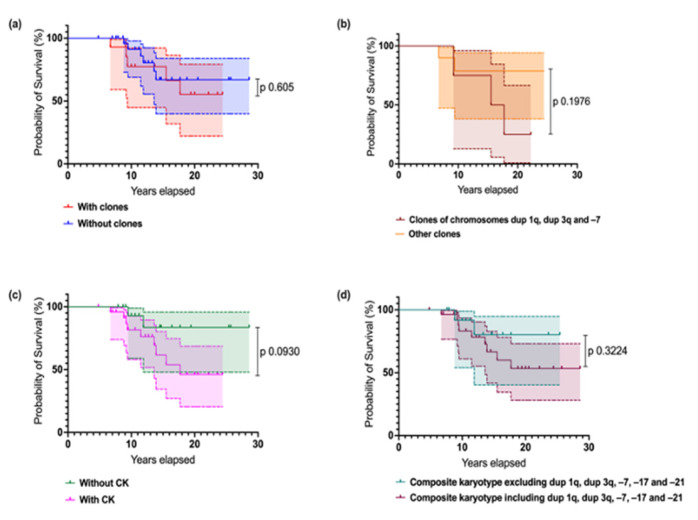
Survival estimates in Mexican individuals with FA according to the presence of the diverse types of cytogenetic abnormalities. (**a**) Survival according to the presence of clonal cytogenetic abnormalities. Patients without clones are in blue, and patients with clones are in red. (**b**) Survival in individuals with cytogenetic clones: aberrations affecting chromosome 1 (1q duplication), 3 (3q duplication), and 7 (7q deletion or –7) in pink, and all other clonal aberrations of indeterminate potential in orange. (**c**) Survival according to the presence of complex karyotypes (CKs): absence of CKs in blue, presence of CKs in red. (**d**) Survival according with the presence of composite karyotypes, including or excluding recognized high-risk CA plus –17 and –21 in FA patients. Log-rank (Mantel-Cox) Test.

Finally, we performed a survival analysis, according to the presence of the diverse types of cytogenetic abnormalities in general and contrasting the presence of the well-known adverse abnormalities in FA (affecting chromosomes 1, 3, and 7) and other clonal chromosome abnormalities of indeterminate potential. In addition, we examined the impact of complex karyotypes upon survival (Figure 9). We did not observe significant differences in patient survival based on the type of CA. However, the presence of clonal cytogenetic abnormalities (CCAs) involving the known high-risk cytogenetic alterations (such as duplication of 1q, duplication of 3q, monosomy 7) complex karyotypes, or composite karyotypes with high-risk alterations plus monosomy 17 and monosomy 21 appears to negatively impact survival in patients with FA. Although these trends were evident, statistical significance may not have been reached, likely due to the small sample size.

## 4. Discussion

In this study, we conducted a cytogenetic cross-sectional analysis of the BM of 43 patients with the chromosome instability and cancer predisposition syndrome, FA. As anticipated, a high number of CAs were observed in virtually all participants. NCCAs were the most prevalent, reflecting an underlying karyotype heterogeneity caused by faulty DNA repair mechanisms in individuals with FA. CCAs, mostly abnormalities of indeterminate potential, were also observed, with high-risk CAs being detected in four patients, two of whom had MDS defining CAs. This comprehensive analysis of the type and frequency of CAs in the BM of individuals with FA stresses the natural history of hematological cancer in FA, from heterogeneous NCCA towards CK, and finally CCA and cancer. This study complements the information on the genomic changes involved in the evolution towards hematological cancer, which has been extensively studied by other authors [22,23,30], given that we analyzed the chromosomes of pre-leukemic BM and at the individual cell level, revealing the presence of composite karyotypes in the BM of practically all patients.

As shown in Figure 3, the observed NCCAs included both structural alterations and aneuploidies (gains and losses of whole chromosomes). Structural alterations included gains of chromosome material and chromosome deletions. Specifically, chromosome gains may arise from the joining of non-homologous chromosome segments through the non-homologous end-joining (NHEJ) pathway, causing chromosome translocations. Additionally, the segregation of radial exchange figures during mitosis could produce daughter cells harboring single derivative chromosomes (with the corresponding gain and/or loss of chromosome material). On the other hand, chromosome deletions were six times more frequent than gains. Their origin could be chromosomal rearrangements or non-repaired double-strand breaks (DSB) that reached mitosis and whose broken segment was not retained in the same cell after exiting mitosis, leaving a deleted chromosome.

We saw an overrepresentation of loss-of-chromosome aneuploidies, raising the possibility of technical errors during the preparation of metaphase spreads; nonetheless, given our strict scoring criteria during chromosome analysis (see methodology), we consider that most of these alterations are genuine chromosome numerical losses. In this context of genomic instability, we hypothesize that chromosome losses are related to the mis-segregation of structurally altered chromosomes that became incorporated into micronuclei and further shattered, as others have shown to occur with lagged chromosomes [41].

Interestingly, we also identified neutral CA, i.e., balanced CA such as chromosome inversions and translocations. Traditionally, these alterations were thought to be less detrimental than deletions and duplications, but it is now recognized that they can have a significant impact in cancer evolution, since the change in the 3D distribution pattern of genes and regulatory elements is responsible for emerging network dynamics [42]. Inversions are not commonly reported in previous cytogenetic studies of patients with FA, most likely because cytogenomic analysis using microarrays is usually preferred over GTG banded chromosomes [23]. These inversions could result from the presence of two DSBs in the same chromosome that were rejoined after a 180-degree rotation. Other mechanisms may also explain the presence of inversions, including chromothripsis-like rearrangements, but with fewer breakpoints than traditional chromothripsis, which usually involves extensive fragmentation and rearrangement of a single chromosome. It is known that an intact FA/BRCA pathway plays a crucial role in the micronucleus-related chromosome fragmentation that leads to chromothripsis, while a deficient FA/BRCA pathway generates significantly less chromosome fragmentation under similar conditions [41]. This type of alteration, evidenced here by G banding, may represent just the tip of the iceberg of more complex ACs; these can be studied at higher resolution, with methodologies such as SKY or M-FISH, with which one can identify CA in greater number and complexity. This undoubtedly deserves further study.

Of note, NCCAs in FA are quite variable from cell to cell, and this is probably due to multiple factors, including the following: first, a continuous generation of DSBs, which are incorrectly and not homogeneously rejoined due to the reliance of the FA cells on error-prone DNA repair pathways [4]; second, dividing cells harboring NCCAs will not parent chromosomally identical daughter cells, particularly when dicentric chromosomes and radial figures are present, as mitotic segregation will not equally distribute the CAs into the daughter cells [2]; third, NCCAs might confer differential surviving capacities, and therefore alterations incompatible with life will not thrive, while fitness-providing CAs will persist, in a constant cycle of cell replacement and cell attrition. During this cycle of depletion and emergence of cells with NCCAs, new genome compositions in the tissue will arise, producing a unique combination of CAs that will benefit the survival of cells harboring stable karyotypes with CCAs.

Cells carrying a specific CCA can be considered evidence of a successful genome arrangement. However, this specific clone could also be transient, as the continuous production of CAs does not cease, leading to the emergence of CKs, most of which are NCCAs. Some CKs may appear in cells that already contain a specific CCA, generating genomes that may either disappear or adapt, eventually evolving towards a clone with higher fitness. In a cell population exhibiting CIN, NCCAs will create dynamic karyotypic diversity. This heterogeneity of cells, with stochastic CAs, eventually gives rise to CCAs, which may be transient, creating a dynamic cycle of NCCA/CCA. The NCCA/CCA cycle establishes a macroevolutive phase that may continue until the emergence of stable clones that encompasses a phase transition. One surviving genome with CCAs carrying high-risk CAs, such as clones with dup(1q), dup(3q), del(7q), and –7, with a composite karyotype showing clonal evolution and a decrease in NCCAs (as in patients with MDS), could be indicative of evolution towards leukemia. Clonal expansion is characteristic for the microevolutionary phase [43]. These specific karyotype alterations are known to facilitate the progression to cancer. The cells with these CAs will proliferate until they become a clone, thus completing the transition from NCCA to CCA. In patients with FA, the transition to cancer also involves specific genetic alterations such as *RUNX1* and *TP53,* among others [23].

Importantly, progression towards cancer, which typically takes a long time in DNA repair-proficient individuals, is accelerated in patients with FA. In this work, through an in-depth cytogenetic analysis of the BM of patients with FA, we recapitulate macroevolution patterns previously described [23,30,31], including an initial phase of high karyotypic heterogeneity leading to more stable “end products of evolution”, i.e., CCA abnormalities that combine large-scale chromosomal changes with genetic mutations and copy number alterations, which will ultimately drive micro-evolution steps towards cancer.

## 5. Conclusions

In this study, we observed that the frequency of ACs was not associated with gender, severity of bone marrow failure, or androgen treatment. The frequency of NCCAs and CCAs increased with age; although no significant correlation was found, a significant difference was observed between older patients with CCAs and younger patients without CCAs.

In general, the preleukemic bone marrow of patients with FA exhibits significant basal karyotypic heterogeneity, evidenced by the widespread presence of NCCAs. This karyotypic heterogeneity precedes the eventual appearance of CKs and the selection of CCA-bearing cells that enhance adaptation, which may be transient until the appearance of a stable CCA. The NCCA/CCA cycle establishes a macroevolutionary phase that could continue until the emergence of stable clones, leading to a phase transition. A surviving CCA genome harboring high-risk ACs emerges, such as clones with dup(1q), dup(3q), del(7q), and –7, and the associated NCCAs decline. These observations fit the model of evolution towards cancer that comprises a first phase of macroevolution represented by NCCA and karyotypic heterogeneity, followed by the establishment of clones with CCAs, which leads to microevolution and cancer.

These observations warrant a longitudinal follow-up study of patients with FA to determine the macro- and microevolutionary phases and to detect potential cytogenetic biomarkers that precede clonal hematopoiesis.

## Figures and Tables

**Table 1:** Clinical characteristics of study population.

Gender and Age of Patients (n = 43)	n	Percentage % or (Range)
Female Male	25 18	58 42
Median age at diagnosis years	8.1	(1.4–15.6)
Median age (years) at cytogenetic study	9.19	(4.7–28.6)
Affected gene (n = 33)		
*FANCA*	22	66.7
*FANCE*	3	9.1
*FANCD2/BRCA1*	1	3.0
*FANCF*	1	3.0
*FANCG*	2	6.1
*FANCJ/BRIP1*	1	3.0
*FANCL*	2	6.1
*FANCN*	1	3.0
BMF Status (n = 43)		
No	5	11.6
Mild	8	18.6
Moderate	15	34.9
Severe	13	30.2
MDS	2	4.7
Androgen treatment (n = 42)		
Yes	29	69
No	13	31
Transfusion dependency (n = 42)		
Yes	13	31
No	29	69
Transplant (n = 42)		
Yes	8	19
No	34	81
Alive/Deceased (Cause of death) (n = 41)		
Alive	30	73.2
Deceased (ALL)	1	2.4
Deceased (BMF)	4	9.8
Deceased (BMF, Intracranial bleeding)	1	2.4
Deceased (HSPCT complication)	4	9.8
MDS	1	2.4

BMF—Bone Marrow Failure. MDS—Myelodysplastic Neoplasm. ALL—Acute Lymphoid Leukemia. HSPCT—Hematopoietic Stem and Progenitor Cell Transplantation.

**Table 2:** Population and clinical data.

ID	Gender	Age at Diagnosis (Years)	Age at Cytogenetic Study (Years)	Affected Gene	BMF STATUS	Androgen Treatment	Transfusion Dependency	HSPCT	Alive/Deceased (Cause of Death)
RAFMex057	Male	4.2	4.7	*FANCE*	Moderate	No	No	No	Alive
RAFMex058	Female	4.1	4.8	NI	Mild	No	No	No	Alive
RAFMex006	Male	3.5	5.5	*FANCF*	Severe	Yes	No	Yes	Alive
RAFMex029	Male	4.5	5.5	NI	Mild	Yes	No	No	Alive
RAFMex009	Female	3.3	6.2	*FANCA*	Moderate	Yes	No	No	Alive
RAFMex043	Male	4.9	6.6	*FANCE*	Severe	Yes	Yes	Yes	Deceased (HSPCT complication)
RAFMex010	Female	5.9	6.7	*FANCA*	Moderate	Yes	No	No	Alive
RAFMex042	Female	6.9	6.9	*FANCL*	Severe	Yes	Yes	Yes	Alive
RAFMex047	Female	7.1	6.9	*FANCA*	Severe	Yes	No	No	Alive
RAFMex002	Female	6.0	7.8	*FANCA*	No	No	No	No	Alive
RAFMex044	Female	1.4	7.9	*FANCA*	Mild	Yes	No	No	Alive
RAFMex033	Male	5.1	8.1	NI	Moderate	No	No	No	Alive
RAFMex025	Female	6.1	8.1	*FANCA*	No	No	No	No	Alive
RAFMex005	Female	6.1	8.2	*FANCA*	Moderate	Yes	No	No	Alive
FANC143	Female	3.6	8.3	*FANCG*	Severe	Unclear	Yes	Yes	Deceased (HSPCT complication)
RAFMex026	Female	8.5	8.5	*FANCE*	Severe	No	No	Yes	Deceased (HSPCT complication)
RAFMex011	Female	7.1	8.7	*FANCA*	Moderate	No	No	Yes	Alive
FANC161	Female	8.0	8.8	NI	Severe	Yes	Yes	NI	Deceased (BMF)
FANC157	Male	9.0	9.0	NI	Severe	Yes	Yes	No	Deceased (BMF)
FANC162	Male	9.1	9.12	NI	Severe	No	NI	No	NI
FANC148	Male	7.7	9.14	NI	Severe	No	Yes	No	Deceased (BMF, Intracranial bleeding)
FANC101	Female	8.2	9.2	*FANCA*	Severe	Yes	Yes	Yes	Deceased (HSPCT complication
RAFMex07	Female	9.7	9.7	*FANCJ/BRIP1*	Moderate	No	No	No	Alive
RAFMex035	Male	9.7	10.2	*FANCA*	Mild	No	No	No	Alive
FANC032	Male	9.2	10.7	*FANCG*	Moderate	Yes	No	No	NI
RAFMex024	Female	8.8	10.9	*FANCA*	Moderate	Yes	Yes	No	Alive
RAFMex08	Male	9.5	11.7	*FANCA*	Mild	Yes	No	No	Deceased (ALL)
RAFMex032	Female	12.6	12.6	NI	Moderate	No	No	No	Alive
RAFMex031	Male	13.1	13.4	*FANCA*	Severe	Yes	Yes	No	Deceased (BMF)
RAFMex018	Female	9.8	14.7	*FANCA*	Severe	Yes	Yes	No	Alive
FANC024	Female	7.4	16.7	*FANCA*	Moderate	Yes	No	Yes	Alive
RAFMex051	Female	11.7	17.7	*FANCA*	Moderate	Yes	Yes	No	Alive
RAFMex036	Male	6.4	17.8	*FANCA*	Moderate	Yes	No	No	Alive
RAFMEX066	Male	9.5	17.9	*FANCL*	No	Yes	No	No	Alive
RAFMex038	Male	12.7	18.4	*FANCA*	Mild	Yes	No	No	Alive
RAFMex037	Female	14.4	20.2	*FANCA*	No	Yes	No	No	Alive
RAFMex059	Male	13.3	22.50	NI	Moderate	Yes	No	No	Alive
RAFMex027	Male	5.6	22.51	*FANCN*	No	No	No	No	Alive
RAFMex022	Female	15.6	25.0	*FANCA*	Mild	Yes	Yes	No	Alive
RAFMex034	Female	11.7	25.7	*FANCA*	Mild	Yes	No	No	Alive
RAFMex030	Female	12.0	28.6	NI	Moderate	Yes	No	No	Alive
FANC031	Female	10.3	15.5	*FANCD2*	MDS	Yes	Yes	No	Deceased (MDS)
RAFMex015	Male	9.7	17.2	*FANCA*	MDS	Yes	Yes	No	Deceased (MDS)

BMF—Bone Marrow Failure. HSPCT—Hematopoietic Stem and Progenitor Cell Transplantation. ALL—Acute Lymphoid Leukemia. MDS—Myelodysplastic Neoplasm. NI—No Information.

**Table 3:** Cytogenetic findings in the BM of 43 patients with FA.

n	ID	Total Frequency of CA	Frequency of NCCA/Cell (Frequency of Cells with CK)		Frequency of CCA/Cell	Involved Chromosome in CCA	Genes Involved in AML and MDS
1	RAFMEX057	0.8	0.55	(0.10)	0.25	del(3q27)	*TERC*
2	RAFMEX058	0.26	0.26	(0.04)	0.00		
3	RAFMEX006	0.75	0.75	(0.04	0.00		
4	RAFMEX029	0.65	0.65	(0.05)	0.00		
5	RAFMEX009	0.45	0.45	(0.00)	0.00		
6	RAFMEX043	0.54	0.33	(0.03)	0.21	del(6p22)	
7	RAFMEX010	0.50	0.30	(0.05)	0.20	–21	*RUNX1*, *U2AF1*, *ERG*
8	RAFMEX042	0.36	0.36	(0.00)	0.00		
9	RAFMEX047	0.00	0.00	(0.00)	0.00		
10	RAFMEX002	0.23	0.00	(0.00)	0.23	add(2p)	
11	RAFMEX044	0.50	0.50	(0.05)	0.00		
12	RAFMEX033	0.90	0.90	(0.21)	0.00		
13	RAFMEX025	0.85	0.85	(0.10)	0.00		
14	RAFMEX005	0.90	0.75	(0.10)	0.15	–20	*ASXL1*
15	FANC143	0.60	0.60	(0.10)	0.00		
16	RAFMEX026	0.95	0.63	(0.11)	0.32	dup(3q26)	*MECOM*, *MDS1* and *EVI1* complex locus, *TERC*
17	RAFMEX011	0.40	0.40	(0.00)	0.00		
18	FANC161	0.35	0.35	(0.05)	0.00		
19	FANC157	0.10	0.10	(0.00)	0.00		
20	FANC162	0.10	0.10	(0.00)	0.00		
21	FANC148	1.25	0.55	(0.15)	0.70	+8	*MYC*
22	FANC101	0.40	0.40	(0.00)	0.00		
23	RAFMEX007	0.85	0.85	(0.10)	0.00		
24	RAFMEX035	0.25	0.25	(0.00)	0.00		
25	FANC32	0.30	0.30	(0.00)	0.00		
26	RAFMEX024	0.15	0.15	(0.00)	0.00		
27	RAFMEX008	0.63	0.63	(0.05)	0.00		
28	RAFMEX032	0.70	0.40	(0.10)	0.30	–17, –22	*TP53*, *NF1*, *PPM1D*, *ERBB2*, *SRSF2*, *PRPF8*, *EP300*
29	RAFMEX031	1.20	1.20	(0.15)	0.00		
30	RAFMEX018	0.05	0.05	(0.00)	0.00		
31	FANC24	0.40	0.25	(0.05)	0.15	–18	*SETBP1*
32	RAFMEX051	0.22	0.22	(0.00)	0.00		
33	RAFMEX036	0.68	0.68	(0.05)	0.00		
34	FANC46	1.13	0.93	(0.07)	0.20	–16	*CREBBP*, *CTCF 8*
35	RAFMEX038	0.81	0.67	(0.00)	0.14	add(3p25)	
36	RAFMEX037	0.99	0.99	(0.06)	0.00		
37	RAFMEX059	0.75	0.50	(0.15)	0.25	der(6)t(1q;6p)	*MDM4*
38	RAFMEX027	0.70	0.45	(0.10)	0.25	del(3p13)	
39	RAFMEX022	0.30	0.30	(0.00)	0.00		
40	RAFMEX034	0.25	0.25	(0.00)	0.00		
41	RAFMEX030	0.35	0.35	(0.05)	0.00		
42	FANC31	1.05	0.45	(0.1)	0.60	–7, –8	*CUX1*, *SAMD9*, *MLL3*, *EZH2*, *EGFR*, *LUC7L2*, *MYC*
43	RAFMEX015	3.40	0.40	(1.00)	3.00	–7, der(18)t(3;18)	*CUX1*, *SAMD9*, *MLL3*, *EZH2*, *EGFR*, *SETBP1*
	SUM (percentage)	27	20.05 (74.3%)		6.95 (25.7%)		
	Average	0.63	0.47	(0.07)	0.16		

Abbreviatures: CA—Chromosome Abnormality. NCCA—Non-Clonal Chromosome Abnormality. CK—Complex Karyotype. CCA—Clonal Chromosome Abnormality.

## Data Availability

The original contributions presented in this study are included in the article/Supplementary Materials. Further inquiries can be directed to the corresponding author.

## Supplementary Materials

The following supporting information can be downloaded at: https://www.mdpi.com/article/10.3390/cancers17111805/s1, Supplementary Table S1. Database containing the cytogenetics of 43 patients with FA. Complete data. Supplementary Table S2. Table containing data on the genes and variants of patients with FA. Supplementary Table S3. Genes located in CCA, involved in MDS and AML [16,17,23,26,44,45,46,47,48,49,50,51,52,53,54,55,56,57,58,59,60,61,62,63,64,65,66,67,68,69].
