# Supplementary material for: High Burden of Non-Clonal Chromosome Aberrations Before Onset of Detectable Neoplasia in Fanconi Anemia Bone Marrow
Source: Cancers (Basel). 2025 May 28;17(11):1805. doi: 10.3390/cancers17111805 (PMC12153895; doi:10.3390/cancers17111805)
Supplement: Supplementary file 1 [file cancers-17-01805-s001.zip › Supplementary Table 2 Round 2.pdf]

Supplementary Table 2. Affected genes, type and classification of the variants found in this group of FA patients.

ID	Affected gene	Zygosity	Allele 1		Allele 2		BMF Status
			Type of variant	ACMG classification	Type of variant	ACMG classification	
RAFMex002	*FANCA*	Heterozygous	Substitution	Likely Pathogenic	Substitution	Pathogenic	No
RAFMex025	*FANCA*	Homozygous	Frameshift	Pathogenic	Frameshift	Pathogenic	No
RAFMex037	*FANCA*	Homozygous	Deletion	Pathogenic	Deletion	Pathogenic	No
RAFMex008	*FANCA*	Homozygous	Deletion	Pathogenic	Deletion	Pathogenic	Mild
RAFMex034	*FANCA*	Homozygous	Deletion	Pathogenic	Deletion	Pathogenic	Mild
RAFMex044	*FANCA*	Homozygous	Deletion	Pathogenic	Deletion	Pathogenic	Mild
RAFMex022	*FANCA*	Homozygous	Missense	Pathogenic	Missense	Pathogenic	Mild
RAFMex035	*FANCA*	Heterozygous	Missense	Pathogenic	Substitution	VUS	Mild
RAFMex038	*FANCA*	Homozygous	Deletion	Pathogenic	Deletion	Pathogenic	Mild
RAFMex024	*FANCA*	Homozygous	Frameshift	Pathogenic	Frameshift	Pathogenic	Moderate
RAFMex010	*FANCA*	Homozygous	Nonsense	Pathogenic	Nonsense	Pathogenic	Moderate
RAFMex005	*FANCA*	Heterozygous	Missense	Pathogenic	Missense	Pathogenic	Moderate
RAFMex009	*FANCA*	Homozygous	Deletion	Pathogenic	Deletion	Pathogenic	Moderate
FANC024	*FANCA*	Heterozygous	Deletion	Pathogenic	Substitution	Pathogenic	Moderate
RAFMex011	*FANCA*	NI	NI	NI	NI	NI	Moderate
RAFMex036	*FANCA*	Heterozygous	Deletion	Pathogenic	Deletion	Pathogenic	Moderate
RAFMex051	*FANCA*	Heterozygous	Missense	Pathogenic	Deletion	Pathogenic	Moderate
RAFMex031	*FANCA*	Homozygous	Deletion	Pathogenic	Deletion	Pathogenic	Severe
RAFMex047	*FANCA*	Heterozygous	Deletion	Pathogenic	Frameshift	Likely Pathogenic	Severe
FANC101	*FANCA*	Homozygous	Frameshift	Pathogenic	Frameshift	Pathogenic	Severe
RAFMex018	*FANCA*	Heterozygous	Frameshift	Pathogenic	Substitution	Pathogenic	Severe
RAFMex015	*FANCA*	Homozygous	Substitution	VUS	Substitution	VUS	**MDS**
RAFMex057	*FANCE*	Homozygous	Missense	Pathogenic	Missense	Pathogenic	Moderate
RAFMex043	*FANCE*	Homozygous	Missense	Pathogenic	Missense	Pathogenic	Severe
RAFMex026	*FANCE*	Homozygous	Missense	Pathogenic	Missense	Pathogenic	Severe
RAFMex006	*FANCF*	Homozygous	Frameshift	Pathogenic	Frameshift	Pathogenic	Severe
FANC032	*FANCG*	Homozygous	Frameshift	Pathogenic	Frameshift	Pathogenic	Moderate
FANC143	*FANCG*	Homozygous	Frameshift	Pathogenic	Frameshift	Pathogenic	Severe
FANC046	*FANCL*	Homozygous	Missense	VUS	Missense	VUS	No
RAFMex042	*FANCL*	Homozygous	Missense	VUS	Missense	VUS	Severe
FANC031	*FANCD2*	Heterozygous	Frameshift	Pathogenic	Missense	VUS	**MDS**
RAFMex07	*FANCJ/BRIP1*	Homozygous	Nonsense	Pathogenic	Nonsense	Pathogenic	Moderate
RAFMex027	*FANCN/PALB2*	Heterozygous	Insertion	Likely Pathogenic	NI	NI	No

ACMG-American College of Medical Genetics, BMF-Bone Marrow Failure, **MDS-** Myelodysplastic Neoplasm. NI-No Information.
