# Supplementary material for: High Burden of Non-Clonal Chromosome Aberrations Before Onset of Detectable Neoplasia in Fanconi Anemia Bone Marrow
Source: Cancers (Basel). 2025 May 28;17(11):1805. doi: 10.3390/cancers17111805 (PMC12153895; doi:10.3390/cancers17111805)
Supplement: Supplementary file 1 [file cancers-17-01805-s001.zip › Supplementary Table 3 fot ProofRead.pdf]

Supplementary Table 3. Genes located in CCA, involved in MDS and AML

Involved chromosome in CCA	Genes located in CCA, Involved in AML and MDS	Gene Function
del(3q27)	*TERC.*	Telomerase, deregulation in somatic cells may be involved in oncogenesis, [1,2]
del(6p22)	*SOX4*	SOX4-induced upregulation of ARHGAP9 promotes the progression of acute myeloid leukemia [3,4].
	KDM1B/LSD2	Histone demethylase, impacts DNA methylation, cancer cell reprogramming, E3 ubiquitin ligase activity and DNA damage repair pathways [5].
–21	*RUNX1*	Transcription factor, regulates hematopoiesis and pro-platelet formation, related with increased risk to develop myeloid malignancies, most commonly MDS, LMA and chronic myelomonocytic leukemia (CMML) [6]. In MDS, mutant RUNX1 attenuates the G1/S cell cycle checkpoint, normally activated in AF in response to DNA damage [7].
–20	*ASXL1*	Mutations of this gene are associated with disease progression from MDS to AML, including clone appeared in late stage of MDS in FA patients [8].
dup(3q26)	*EVI1*	Transcriptional regulator and oncoprotein that may be involved in hematopoiesis, apoptosis, development, and hereditary hematological neoplasia [9]. Overexpression might confer clonal advantage in FA [2]
+8	*MYC*	Proto-oncogene, amplification of this gene has been reported in 1% of the MDS and AML cases, is associated with oncogenic progression from MDS to AML transformation [10] [11]. In FA is involved in the proliferation of hematopoietic stem cells [12].
–17	*TP53*	Transcription factor and tumor suppressor, is commonly mutated in myeloid neoplasms patients, closely related to complex karyotypes and secondary AML developed from MDS [13]. Highly expressed in FA hematopoietic stem and progenitor cells [14].
	NF1	Tumor suppressor gene, negative regulator of the reticular activating system (RAS) signal transduction pathway, constitutional mutations predispose to myeloid malignancies such as AML, changes in this gene are present in 0 to 9% of MDS [15].
	PPM1D	The expression of this gene is induced in a p53-dependent manner in response to various environmental stresses, variants in this gene participates in the clonal expansion in myeloid malignancies [16].
	ERBB2	Member of the epidermal growth factor (EGF), overexpression of this gene has been reported in numerous cancers [17].
	SRSF2	Part of the spliceosome, mutations in SRSF2 in MDS predicted shorter overall survival and more frequent AML [18].
–22	*EP300*	Important in pathways like tumor suppressor and pro-oncogenic processes, because of their roles in regulation of cell cycle and proliferation [19].
–18	*SETBP1*	Its protein binds directly with the SET nuclear protein. Its overexpression or deletion is associated with poor prognosis in MDS or AML and dysregulation in normal or malignant hematopoiesis [20].
–16	*CREBBP*	Paralog of *EP300;* regulation of cell cycle and proliferation [19].
	CTCF	Have a key role in chromatin structure, mutations in this gene have been associated with invasive breast cancers, prostate cancers, and Wilms' tumors [21].
der(6)t(1q;6p)	*MDM4*	Regulator of p53 (1q) drives clonal hematopoiesis, leading to a series of steps that will eventually reduce TP53 expression in FA [22].
del(3p13)	*FOXP1*	Upregulates the cell stress sensor SIRT1 and supports AML cell resistance to therapeutic treatments, also limits superoxide anion levels and oxidative stress of myeloid leukemia cells [23].
	PPP4R2	Required for efficient DNA double strand break repair, is recurrently deleted in acute myeloid leukemia [24].
–7	*CUX1*	Play a role in the cell cycle progression and has a tumor suppressor activity in myeloid cells [25].
	SAMD9	Is an interferon responsive antiviral gene. Its mutation causes severe pancytopenia and predisposition to BMF and MDS. SAMD9/SAMD9L mutations, are among the most frequent germline mutations in pediatric MDS [6,26]
	MLL3	Promotes leukemogenesis, with most frequent events in human cancer [27].
	EZH2	Is a histone methyltransferase that can silence genes involved in cell cycle, differentiation and cell proliferation, its deficiency is related with the transformation from MDS to AML [28]
	EGFR	Is part of the protein kinase superfamily, key regulator in cell proliferation, differentiation, division, survival, and cancer development, it is a therapeutic target for tyrosine kinase inhibitors in MDS and AML cells [29]
	LUC7L2	Splicing factor involved in the evolution of MDS to AML, alters the hematopoietic differentiation of induced stem cells [18,30].

Supplementary Table 3 references

1. Schratz, K.E.; Gaysinskaya, V.; Cosner, Z.L.; DeBoy, E.A.; Xiang, Z.; Kasch-Semenza, L.; Florea, L.; Shah, P.D.; Armanios, M. Somatic reversion impacts myelodysplastic syndromes and acute myeloid leukemia evolution in the short telomere disorders. *J. Clin. Invest.* **2021**, *131*, 1–9.
2. Quentin, S.; Cuccuini, W.; Ceccaldi, R.; Nibourel, O.; Pondarre, C.; Pagès, M.P.; Vasquez, N.; D'Enghien, C.D.; Larghero, J.; De Latour, R.P.; et al. Myelodysplasia and leukemia of fanconi anemia are associated with a specific pattern of genomic abnormalities that includes cryptic RUNX1/AML1 lesions. *Blood* **2011**, *117*, 161–170.
3. He, X.; Zou, H.; Wang, F. SOX4-induced upregulation of ARHGAP9 promotes the progression of acute myeloid leukemia. *Drug Dev. Res.* **2021**, *82*, 1227–1234.
4. Gou, J.; Bi, J.; Wang, K.; Lei, L.; Feng, Y.; Tan, Z.; Gao, J.; Song, Y.; Kang, E.; Guan, F.; et al. O-GlcNAcylated FTO promotes m6A modification of SOX4 to enhance MDS/AML cell proliferation. *Cell Commun. Signal.* **2025**, *23*, 43.
5. Kim, H.M.; Liu, Z. LSD2 Is an Epigenetic Player in Multiple Types of Cancer and Beyond. *Biomolecules* **2024**, *14*.
6. Rudelius, M.; Weinberg, O.K.; Niemeyer, C.M.; Shimamura, A.; Calvo, K.R. The International Consensus Classification (ICC) of hematologic neoplasms with germline predisposition, pediatric myelodysplastic syndrome, and juvenile myelomonocytic leukemia. *Virchows Arch.* **2023**, *482*, 113–130.
7. Marion, W.; Koppe, T.; Chen, C.C.; Wang, D.; Frenis, K.; Fierstein, S.; Sensharma, P.; Aumais, O.; Peters, M.; Ruiz-Torres, S.; et al. RUNX1 mutations mitigate quiescence to promote transformation of hematopoietic progenitors in Fanconi anemia. *Leukemia* **2023**, *37*, 1698–1708.
8. Chang, L.; Cui, Z.; Shi, D.; Chu, Y.; Wang, B.; Wan, Y.; Ma, Q.; Zhang, R.; Li, H.; Cheng, X.; et al. Polyclonal evolution of Fanconi anemia to MDS and AML revealed at single cell resolution. *Exp. Hematol. Oncol.* **2022**, *11*, 1–14.
9. Ottema, S.; Mulet-Lazaro, R.; Beverloo, H.B.; Erpelinck, C.; van Herk, S.; van der Helm, R.; Havermans, M.; Grob, T.; Valk, P.J.M.; Bindels, E.; et al. Atypical 3q26/MECOM rearrangements genocopy inv(3)/t(3;3) in acute myeloid leukemia. *Blood* **2020**, *136*, 224–234.
10. Gajzer, D.; Logothetis, C.N.; Sallman, D.A.; Calon, G.; Babu, A.; Chan, O.; Vincelette, N.D.; Volpe, V.O.; Al Ali, N.H.; Basra, P.; et al. MYC overexpression is associated with an early disease progression from MDS to AML. *Leuk. Res.* **2021**, *111*, 1–17.
11. Huh, Y.O.; Tang, G.; Talwalkar, S.S.; Khoury, J.D.; Ohanian, M.; Bueso-Ramos, C.E.; Abruzzo, L. V. Double minute chromosomes in acute myeloid leukemia, myelodysplastic syndromes, and chronic myelomonocytic leukemia are associated with micronuclei, MYC or MLL amplification, and complex karyotype. *Cancer Genet.* **2016**, *209*, 313–320.
12. Rodrıguez, A.; Zhang, K.; Parmar, K.; Andrea, A.D.D. Clinical and Translational Report MYC Promotes Bone Marrow Stem Cell Dysfunction in Fanconi Anemia Clinical and Translational Report MYC Promotes Bone Marrow Stem Cell Dysfunction in Fanconi Anemia. **2021**, 1–15.
13. Zhao, Y.; Chen, W.; Yu, J.; Pei, S.; Zhang, Q.; Shi, J.; Huang, H.; Zhao, Y. TP53 in MDS and AML: Biological and clinical advances. *Cancer Lett.* **2024**, *588*, 216767.

14. Ceccaldi, R.; Parmar, K.; Mouly, E.; Delord, M.; Kim, J.M.; Regairaz, M.; Pla, M.; Vasquez, N.; Zhang, Q.S.; Pondarre, C.; et al. Bone marrow failure in fanconi anemia is triggered by an exacerbated p53/p21 DNA damage response that impairs hematopoietic stem and progenitor cells. *Cell Stem Cell* **2012**, *11*, 36–49.
15. Philpott, C.; Tovell, H.; Frayling, I.M.; Cooper, D.N.; Upadhyaya, M. The NF1 somatic mutational landscape in sporadic human cancers. *Hum. Genomics* **2017**, *11*, 1–19.
16. Miller, P.G.; Sperling, A.S.; Mayerhofer, C.; McConkey, M.E.; Ellegast, J.M.; Da Silva, C.; Cohen, D.N.; Wang, C.; Sharda, A.; Yan, N.; et al. PPM1D modulates hematopoietic cell fitness and response to DNA damage and is a therapeutic target in myeloid malignancy. *Blood* **2023**, *142*, 2079–2091.
17. Kam, A.Y.F.; Piryani, S.O.; Lee, C.L.; Rizzieri, D.A.; Spector, N.L.; Sarantopoulos, S.; Doan, P.L. Selective ERBB2 and BCL2 inhibition is synergistic for mitochondrial-mediated apoptosis in MDS and AML cells. *Mol. Cancer Res.* **2021**, *19*, 886–899.
18. Douet-Guilbert, N.; Soubise, B.; Bernard, D.G.; Troadec, M.B. Cytogenetic and Genetic Abnormalities with Diagnostic Value in Myelodysplastic Syndromes (MDS): Focus on the Pre-Messenger RNA Splicing Process. *Diagnostics* **2022**, *12*.
19. Attar, N.; Kurdistani, S.K. Acetyltransferases by Cancer. *Cold Spring Harb Perspect Med* **2017**, *7*, a026534.
20. Tanaka, A.; Nishimura, K.; Saika, W.; Kon, A.; Koike, Y.; Tatsumi, H.; Takeda, J.; Nomura, M.; Zang, W.; Nakayama, M.; et al. SETBP1 is dispensable for normal and malignant hematopoiesis. *Leukemia* **2023**, *37*, 1802–1811.
21. Debaugny, R.E.; Skok, J.A. CTCF and CTCFL in cancer. *Curr. Opin. Genet. Dev.* **2020**, *61*, 44–52.
22. Sebert, M.; Gachet, S.; Leblanc, T.; Rousseau, A.; Bluteau, O.; Kim, R.; Ben Abdelali, R.; Sicre de Fontbrune, F.; Maillard, L.; Fedronie, C.; et al. Clonal hematopoiesis driven by chromosome 1q/MDM4 trisomy defines a canonical route toward leukemia in Fanconi anemia. *Cell Stem Cell* **2023**, *30*, 153-170.e9.
23. Levavasseur, F.; Oussous, S.; Zubaidan, T.; Kosmider, O.; Pendino, F.; Rombaut, D.; Bouscary, D.; Fontenay, M.; Lauret, E.; Dusanter-Fourt, I. FOXP1 regulates oxidative stress, SIRT1 expression, and resistance to chemotherapies in acute myeloid leukemia cells. *Blood Adv.* **2023**, *7*, 3265–3275.
24. Herzig, J.K.; Bullinger, L.; Tasdogan, A.; Zimmermann, P.; Schlegel, M.; Teleanu, V.; Weber, D.; Rücker, F.G.; Paschka, P.; Dolnik, A.; et al. Protein phosphatase 4 regulatory subunit 2 (PPP4R2) is recurrently deleted in acute myeloid leukemia and required for efficient DNA double strand break repair. *Oncotarget* **2017**, *8*, 95038–95053.
25. Pellagatti, A.; Boultwood, J. The molecular pathogenesis of the myelodysplastic syndromes. *Eur. J. Haematol.* **2015**, *95*, 3–15.
26. Yoshida, M.; Tanase-Nakao, K.; Shima, H.; Shirai, R.; Yoshida, K.; Osumi, T.; Deguchi, T.; Mori, M.; Arakawa, Y.; Takagi, M.; et al. Prevalence of germline GATA2 and SAMD9/9L variants in paediatric haematological disorders with monosomy 7. *Br. J. Haematol.* **2020**, *191*, 835–843.
27. Chen, C.; Liu, Y.; Rappaport, A.R.; Kitzing, T.; Schultz, N.; Zhao, Z.; Shroff, A.S.; Dickins, R.A.; Vakoc, C.R.; Bradner, J.E.; et al. MLL3 is a haploinsufficient 7q tumor suppressor in acute myeloid leukemia. *Cancer Cell* **2014**, *25*, 652–665.
28. Zheng, Z.; Wang, W.; Feng, M.; Chen, X.; Ren, F.; Hou, Y. The mechanism of

EZH2/H3K27me3 downregulating CXCL10 to affect CD8+ T cell exhaustion to participate in the transformation from myelodysplastic syndrome to acute myeloid leukaemia. *Br. J. Haematol.* **2025**, 1–15.

29. Lainey, E.; Thépot, S.; Bouteloup, C.; Sébert, M.; Ads, L.; Tailler, M.; Gardin, C.; De Botton, S.; Baruchel, A.; Fenaux, P.; et al. Tyrosine kinase inhibitors for the treatment of acute myeloid leukemia: Delineation of anti-leukemic mechanisms of action. *Biochem. Pharmacol.* **2011**, *82*, 1457–1466.
30. Hershberger, C.E.; Moyer, D.C.; Adema, V.; Kerr, C.M.; Hutter, S.; Meggendorfer, M.; Baer, C.; Kern, W.; Nadarajah, N.; Twardziok, S.; et al. HHS Public Access. **2021**, *35*, 1108–1120.
